# Investigating epigenetic effects of activation-induced deaminase in chronic lymphocytic leukemia

**DOI:** 10.1371/journal.pone.0208753

**Published:** 2018-12-20

**Authors:** Maria Schubert, Hubert Hackl, Franz Josef Gassner, Richard Greil, Roland Geisberger

**Affiliations:** 1 Department of Internal Medicine III with Haematology, Medical Oncology, Haemostaseology, Infectiology and Rheumatology, Oncologic Center, Salzburg Cancer Research Institute—Laboratory for Immunological and Molecular Cancer Research (SCRI-LIMCR), Paracelsus Medical University, Salzburg, Austria, Cancer Cluster Salzburg, Salzburg, Austria; 2 Biocenter, Division of Bioinformatics, Medical University of Innsbruck, Innsbruck, Austria; Chang Gung University, TAIWAN

## Abstract

Activation induced deaminase (AID) has two distinct and well defined roles, both relying on its deoxycytidine (dC) deaminating function: one as a DNA mutator and another in DNA demethylation. In chronic lymphocytic leukemia (CLL), AID was previously shown to be an independent negative prognostic factor. While there is substantial impact on DNA mutations, effects of AID on gene expression by promoter demethylation of disease related target genes in leukemia has not been addressed. To shed light on this question, we aimed at determining genome wide methylation changes as well as gene expression changes in response to AID expression in CLL. Although we found minor differences in individual methylation variable positions following AID expression, we could not find recurrent methylation changes of specific target sites or changes in global methylation.

## Introduction

Activation induced deaminase (AID, encoded by the *AICDA* gene) was identified as a key enzyme responsible for somatic hypermutation (SHM) and class switch recombination (CSR) of antibody genes in germinal center B cells [1]. These on-target activities of AID are achieved by deaminating cytosines to uracils at the genomic antibody locus. This initiates an error prone repair process resulting in localized hypermutation in the antigen binding variable region and altered antibody affinity during SHM. During CSR, altered antibody effector function is accomplished through generation of double strand breaks at antibody switch regions [2].

Aside of these on-target activities, AID was also shown to perform substantial off-target effects by mediating genome wide mutations (off-target hypermutation) and translocations (off-target CSR) [3]. These off-target effects significantly contribute to lymphomagenesis as well as to clonal evolution and treatment resistance [4, 5]. Apart from these well described mutagenic effects, AID was also shown to contribute to DNA demethylation by deaminating methylated cytosines, thereby generating thymines, which are eventually replaced by unmethylated dCs by the DNA mismatch repair machinery independent of proliferation or DNA replication [6]. These AID specific epigenetic effects were initially described during reprogramming of germ cells and induced pluripotent stem cells, but were also described for breast cancer, where AID was shown to regulate expression of specific genes important for epithelial-mesenchymal transition [7, 8]. More recently, methylation dynamics during germinal center formation was also attributed to AID activity [9]. In chronic lymphocytic leukemia (CLL), a chronic B cell malignancy of the elderly, AID transcripts in leukemic cells from peripheral blood are detectable in about half of the patients [10]. Patients with AID-expressing cells have a markedly shorter time to treatment, a worse clinical prognosis and more adverse cytogenetic aberrations [11, 12]. Surprisingly, AID expression also correlates with expression of a non-hypermutated B cell receptor (BCR) and is associated with active CSR and an increased proliferative and antiapoptotic potential in a subpopulation of leukemic cells, whereas CLL samples carrying a hypermutated BCR frequently lack AID transcripts [12, 13]. However, although there is an inverse correlation between AID expression and mutated BCRs in CLL, BCR mutation status and AID expression remain both independent parameters for shorter time to treatment in multivariate analyses [12]. CLL samples were also shown to exhibit diverse methylation patterns with high interpatient heterogeneity and distinct methylation dynamics during disease progression [14, 15]. While substantial impact of AID on mutations in CLL was suggested [16], up to now, there are no studies addressing a possible involvement of AID in particular epigenetic changes in CLL.

## Materials and methods

### Patient samples and plasmids

Peripheral blood mononuclear cells from CLL patients (S1 Table) were obtained upon informed consent and ethical approval by the Ethics Committee of the Province of Salzburg (415-E/1287/4–2011, 415-E/1287/8–2011) by Ficoll density gradient centrifugation. The determination of prognostic markers and FISH analysis for trisomy 12, del11q, del13q, and del17p was performed routinely at our department as described previously [17].

Plamids pGFP and pAID-GFP were constructed as previously described [18].

### Realtime RT-PCR

Total RNA was isolated from PBMCs from CLL samples and the Mec1 cell line as a positive control using RNeasy Mini Kit (Cat-No. 74106; QIAGEN) according to the manufacturer’s instructions (no DNA digestion step). 500 ng RNA was reversed transcribed in 20 μl using the iScript cDNA Synthesis Kit (Cat-No. 1708891; BIORAD) with 25°C for 5 minutes, 42°C for 30 minutes and heated to 85°C for 5 minutes. Samples were diluted to a final volume of 100 μl using NF ddH2O. For TaqMan-based realtime RT-PCRs aliquots of 0,4 μl were used in 20 μl final reaction volume and gene expression levels of AICDA (Hs00757808_m1) and 18S rRNA as a control (Hs03928985_g1) were determined on a ViiA 7 Real-Time PCR System (Thermo Fisher Scientific) as follows: 95°C for 10 min, 50 cycles with 95°C for 15 sec, 60°C for 1 min. Samples with Cт values above 32 were considered as non-AID expressors.

### Transfection of human PBMCs for MethEPIC

Fresh human PBMCs (≥ 89% B-CLL cells) of CLL patients (#1, #2, #3 and #4) were transfected with pAID-GFP or pGFP using the Amaxa Human B Cell Nucleofector Kit (Cat. No. VPA-1001; Lonza) adapted from the manufacturer’s instructions. Briefly, 75 x 10^6^ primary CLL cells were transfected with 30 μg of the plasmid (0,4 μg/mio). The nucleofector program U-015 was used. Cells were cultured in RPMI supplemented with 10% (v/v) fetal bovine serum, 1% (v/v) PenStrep (100 U/ml penicillin and 100 μg/ml streptomycin) and 2 mM L-glutamine at 37°C in a 5% CO2 humified incubator for 24 h. Cells were stained for CD5+/CD19+ CLL cells (antibodies) prior sorting of CD5+/CD19+/GFP+ cells on a FACS AriaIII system (Beckton Dickinson) with a purity of 96–99% followed by isolation of genomic DNA using the QIAamp DNA Mini Kit (Cat-No:51306; QIAGEN). 500 ng genomic DNA (10 ng/μl) of each sample was subjected to genome-wide methylation analysis on an Infinium MethylationEPIC BeadChip (Illumina). The data of the Infinium MethylationEPIC BeadChip array are available on ArrayExpress (www.ebi.ac.uk/arrayexpress) under accession number E-MTAB-7431.

### Bioinformatics

Data of the Infinium MethylationEPIC BeadChip (Illumina) were analyzed with the Chip Analysis Methylation Pipeline (ChAMP), adapted for paired analyses using R/Bioconductor, including beta-mixture quantile normalization (BMIQ), linear models and empirical bayes methods (limma) for significant methylation variable positions (MVP) (p<0.05 or p<0.001 as stated) [19]. These MVPs were further grouped into differentially methylated regions (DMRs) defined as regions with 3 MVP in 1000 bp with p<0.05. Adjusted p-values were calculated based on the false discovery rate (FDR) according to the Benjamini-Hochberg method.

### TaqMan gene expression assays (384-well microfluidic cards)

For transfected samples, frozen human PBMCs (≥ 90% B-CLL cells) of patient CLL 116, CLL 237, CLL 790, CLL 857, CLL 660 and CLL 697 were thawed and transfected with pAID-GFP or pGFP using the Amaxa Human B Cell Nucleofector Kit (Cat. No. VPA-1001; Lonza) adapted from the manufacturer’s instructions and identically to the first transfection. For sorting the cells were stained for CD5+/CD19+ (antibodies) and sorted on a FACS AriaIII system (Beckton Dickinson) with a purity of 96,6–100% followed by isolation of RNA with High Pure RNA Isolation Kit (DNase digestion step included) (Cat-No: 11 828 665 001; Roche).

For patient screening, frozen human PBMCs (≥ 90% B-CLL cells) were thawed and purified untouched using MACS Kit (Cat-No: 130-103-466; Miltenyi Biotec), stained for purity check with CD5+/CD19+ (antibodies) revealing a purity of 94,5–98,2% followed by isolation of RNA with High Pure RNA Isolation Kit (DNase digestion step included) (Cat-No: 11 828 665 001; Roche).

For gene expression assays on 384-Well Microfluidic Cards, 150 ng RNA of transfected samples and 200 ng RNA of untransfected samples (patient screening) was reversed transcribed in 20 μl using the iScript cDNA Synthesis Kit (Cat-No. 1708891; BIORAD) with 25°C for 5 minutes, 42°C for 30 minutes and heated to 85°C for 5 minutes. Samples were diluted to a final volume of 55 μl using NF ddH2O and mixed with 55 μl TaqMan Gene Expression Master Mix (Cat-No: 4369016; Applied Biosystems). 100 μl per sample were subjected to TaqMan Gene Expression Assays according to the manufacturer’s instructions, 8 samples per card. Samples were analyzed on a ViiA 7 Real-Time PCR System (Thermo Fisher Scientific) as follows: 95°C for 10 min, 50 cycles with 95°C for 15 sec, 60°C for 1 min. For transfected samples the transcript levels were normalized to GAPDH and the relative fold change was calculated with the 2 ^-ΔΔCT^ method using the pGFP transfected condition as the calibrator.

For untransfected samples the transcript levels were normalized to GAPDH and the relative expression was calculated with the 2 ^-ΔCT^ method. Samples with “Undetermined” Cт values for AICDA were considered negative for AID expression and grouped accordingly. Target genes below the detection limit with “Undetermined” Cт values were artificially set to 10−^11^ (n.d. = not detected).

### Western blotting

Western blots were performed on cell lysates of unsorted pAID-GFP or pGFP transfected human PBMCs (≥ 89% B-CLL cells) with antibodies specific for GFP (#2555, Cell Signaling) and Pan-Actin (D18C11, #8456, Cell Signaling).

### Bacterial mutator assay

AID-GFP fusion gene or GFP of our CLL transfection vectors were PCR-amplified and cloned into a pTrc99 backbone. pTrc99-AID was used as a positive control. Bacterial assays were performed as previously described [20]. The Uracil-DNA-Glycosylase (UNG)-deficient *E*.*coli* strain BW310 was transformed with pTrc99-AID, pTrc99-AID-GFP or pTrc99-GFP. Upon AID-induced mutations of the Rifampicin (Rif)-sensitive allele in BW310 bacteria, Rif-resistant clones appear in presence of Rif. The mutational frequency was determined by plating cultures of individual clones grown over night at 30°C in rich medium supplemented with Carbenicillin (CB) (100 μg/ml) and IPTG (1mM) on LB agar supplemented with CB (100 μg/ml) or CB (100 μg/ml) and Rif (100 μg/ml). AID activity was analyzed by calculating the mutational frequency as ratio of Rif-resistant clones to total clones in the starting culture.

## Results

**Fig 1:**
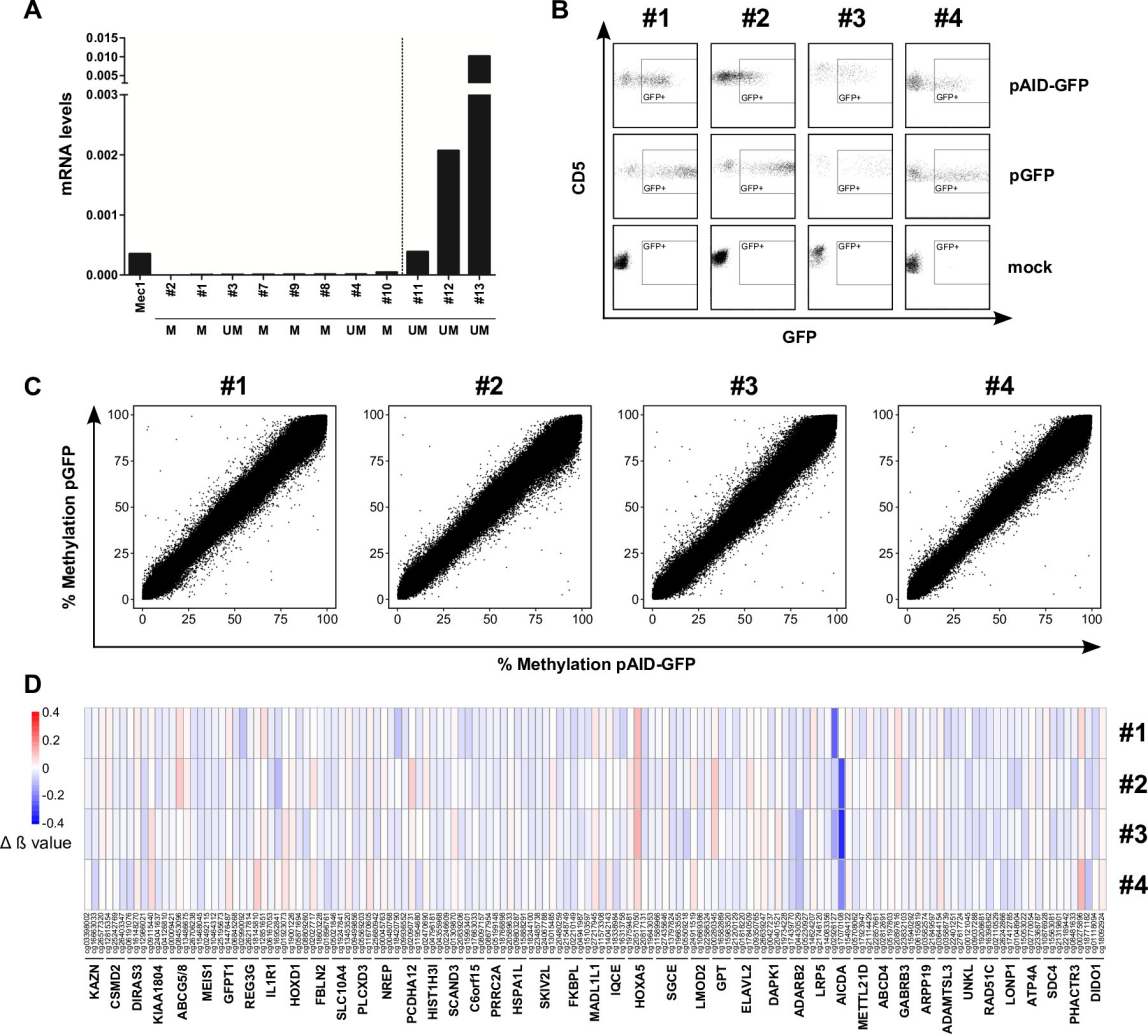
Influence of AID expression on dC-methylation in primary CLL patients. **(A)** Bars show relative AID mRNA levels (normalized to 18S rRNA) of 11 purified CLL samples (patient information on S1 Table) and Mec1 cells detected by realtime RT-PCR. The dashed line divides non-AID expressors (left) and AID expressors (right). M = IGHV mutated; UM = IGHV unmutated. **(B)** 4 non-AID expressors (#1, #2, #3, #4) were either mock transfected or transfected with pGFP or pAID-GFP. FACS plots show gating of GFP+ CD5+ CLL cells after doublet discrimination for cell sorting. **(C)** Scatter plots show percent DNA methylation of 802,850 CpG sites detected with EPIC arrays of the 4 non-AID expressors shown in **B** transfected with pGFP versus pAID-GFP. **(D)** Differences in DNA methylation upon AID expression (delta beta value; red = hypermethylated, blue = hypomethylated) are shown as heatmap of the 145 most confident MVPs (including cg-ID) grouped into 45 DMRs. The respective target genes are indicated below.

To draw light on the question of involvement of AID in epigenetic changes in CLL, we performed methylation array analyses using Illumina Infinium Methylation EPIC BeadChips to assess methylation changes in primary AID non-expressing CLL samples upon transfection with AID encoding constructs. Therefore, we first selected four AID non-expressing CLL samples (#1, #2, #3, and #4) and transfected cells individually with constructs encoding a functional AID-GFP fusion protein or a GFP control (activity of the AID-GFP fusion protein is determined by bacterial mutator assays, S1 Fig). In order to show expression of the AID-GFP fusion protein, we performed western blot experiments on cell lysates from transfected unsorted CLL samples (S2 Fig). For methylation array analyses we sorted cells according to GFP fluorescence and CD5+CD19+ expression by flow cytometry (Fig 1A and 1B) and performed individual EPIC array analysis on DNA from AID-GFP and GFP transfected samples (Fig 1C). No significant global methylation changes were detectable in GFP versus AID-GFP transfected samples, reflected in similar methylation values within the respective CpG regions between GFP and AID-GFP expressing samples (S2 Table). The analysis of individual methylation variable positions (MVPs) between paired AID-GFP and GFP samples revealed only minor differences, with methylation changes at most +/- 20%. In total, we detected 34,868 MVPs with p<0.05 (16,135 hypermethylated and 18,733 hypomethylated MVPs) within or in proximity to 12,727 genes and 293 MVPs with p<0.001 (151 hypermethylated and 142 hypomethylated MVPs) with 184 related genes. Five of these MVPs mapped to the gene body of *AICDA*, most likely coming from the transfected AID encoding plasmid (S3 Table). However, none of these MVPs (except one for *AICDA*) were significantly deregulated using adjusted p-values (false detection rates <0.05, S3 Table).

To determine whether the minor methylation changes detected upon AID expression lead to an altered gene expression, we first ranked the most likely candidate genes with recurrently altered CpG methylation. Therefore, we grouped MVPs into differentially methylated regions (DMRs), defined as regions with at least 3 MVPs within a region of 1kb with p<0.05, resulting in a list of 125 DMRs containing 396 MVPs (S4 Table). Upon exclusion of pseudogenes and inclusion of DMRs harboring at least 2 hypomethylated MVPs located within 1500 bp upstream of the transcription start site (TSS), the gene body or 5′-UTR/3′-UTRs, we ended up with a list of 45 target genes (Fig 1D). For each patient, differences in DNA methylation upon AID expression (delta beta value) of the 145 MVPs grouped into 45 DMRs are shown in Fig 1D.

**Fig 2:**
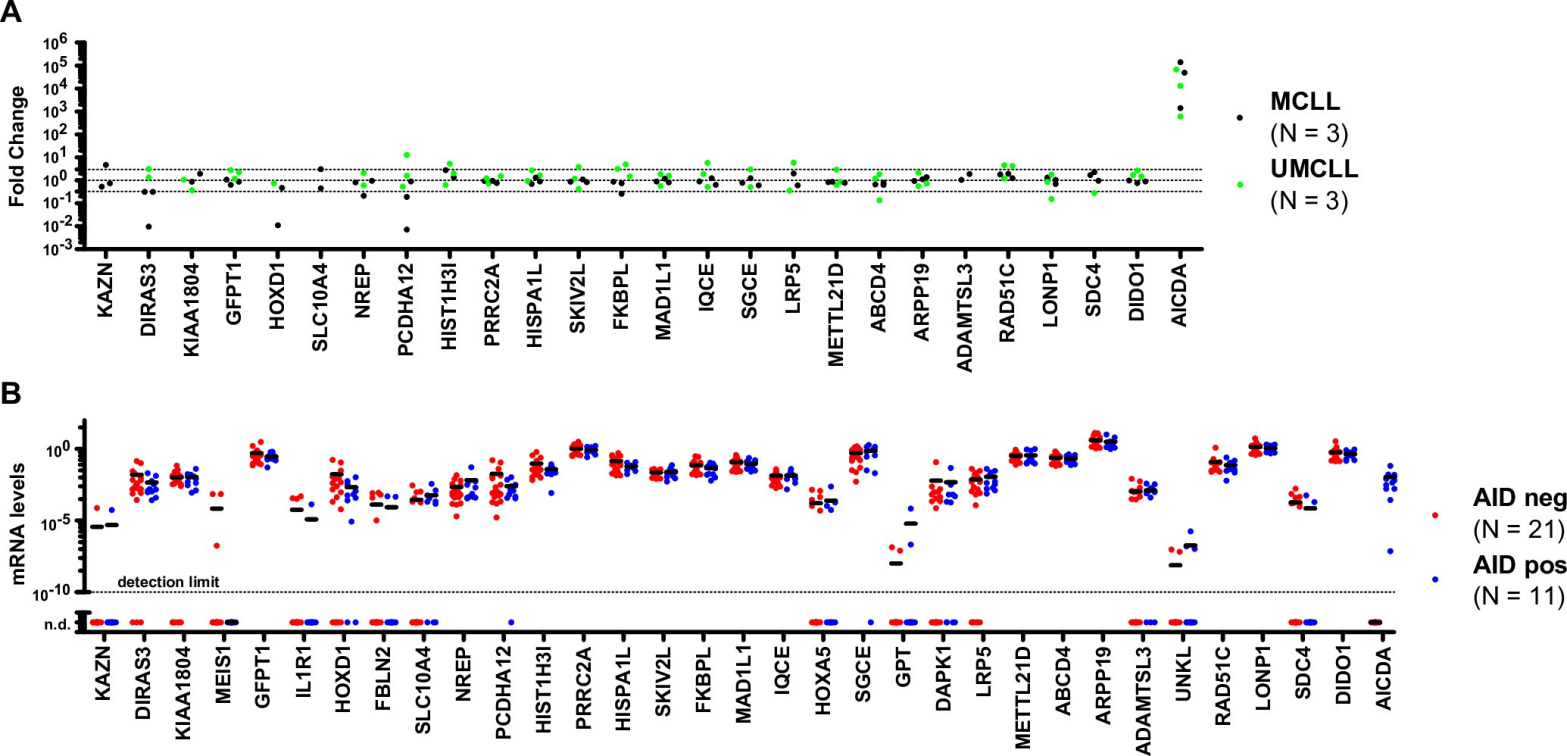
Impact of AID on target gene expression. **(A)** Differential target gene mRNA expression of 6 non-AID expressors transfected with pGFP or pAID-GFP are shown. AICDA served as an internal control. Transcript levels were normalized to GAPDH and the relative fold changes are shown (2 ^-ΔΔCT^ method). The dashed lines show 3-fold increase or decrease in expression. M = IGHV mutated; UM = IGHV unmutated. From the 45 target genes, only the 26 genes with transcripts detectable at least in one sample are given on the x-axis. Samples without detectable expression of the respective gene are not shown. **(B)** Target gene mRNA levels of CLL cells from 32 CLL patients (AID neg = non AID-expressors; AID pos = AID expressors) detected by realtime RT-PCR are shown relative to GAPDH (2 ^-ΔCT^ method). The dashed line represents the detection limit (n.d. = not detected). From the 45 target genes, only 33 genes showed detectable expression and are listed on the x-axis.

To assess whether any of these 45 genes exhibit differential gene expression dependent on AID, we transfected 6 AID non-expressing CLL samples with constructs encoding AID-GFP or GFP and extracted total RNA from GFP positively sorted cells. Reversely transcribed complementary DNA of these samples were subjected to TaqMan 384-well microfluidic cards designed to detect specific transcription levels of our 45 candidate genes in addition to *AICDA*. In accordance with the overall low methylation changes, our results did not reveal a significant impact of AID on differential gene expression and only minor differences in transcription levels could be detected. Of note, only 26 of our set of 45 genes showed detectable expression. Differential expression of *AICDA* served as an internal control (Fig 2A). Using the Taqman microfluidic cards, we also analyzed a cohort of 32 unselected untreated CLL patients (patient information in S1 Table) for expression of *AICDA* and our set of candidate genes. As shown in Fig 2B, *AICDA* expression in 21 of 32 patients (65.6%) was below detection limit and again no differential expression of any of these genes could be noticed in dependence of AID expression.

To address differential gene expression in a larger cohort, we finally reanalyzed a published gene expression dataset (GSE39671) [21]. These microarray expression data from 130 CLL samples revealed bimodal gene expression of *AICDA*, dividing samples into 91 *AICDA* non- (or low)-expressing samples and 39 *AICDA*-expressing samples (S5 Table). In this large cohort, we found five of our 45 target genes to be differentially expressed (FDR<0.05; *GABRB3*, *GFPT1*, *UNKL*, *GPT*, *DAPK1*) in AICDA-high vs low groups, albeit at very low fold change (S5 Table, S3 Fig) and partially with inverse fold change compared to our results from the TaqMan 384-well microfluidic cards (Fig 2B). In addition, differential expression of these candidates could not be validated using another dataset (GSE22762) from CLL samples [22]. In this dataset, *AICDA* expression was not bimodal but rather exhibited a range from low to high (S6 Table) with no apparent correlation (R^2^ range: 0,0001–0,055) with any of our candidate genes (S7 Table).

## Conclusion

We conclude that unlike to data from breast cancer, where AID was recently shown to robustly induce gene expression of genes important for epithelial-mesenchymal transition [8], we could not find comparably convincing and robust AID dependent induction of target gene demethylation and concurrent gene expression in CLL. While we defined a small set of genes with AID-dependent methylation differences, we were unable to detect substantial influence on gene expression differences of these candidate genes, neither in our own cohort of CLL samples nor in data from published datasets [21, 22]. Hence, we assume that possibly either AID does not have a particular set of specific target genes for demethylation in CLL or that there is a high interpatient target heterogeneity, implying that AID–if at all—rather unspecifically induces genome wide ‘off-target’ methylation changes. Alternatively, AID could induce methylation changes in CLL, which were not covered by the EPIC beadchip array or transcriptome analysis, which could affect genome instability rather than gene expression changes. Furthermore, the possibility that we missed single CpGs influencing gene expression, especially in promotor region, by grouping at least 3 MVPs within a region of 1kb to DMRs cannot be excluded.

Summarizing, our results suggest that the well described mutagenic effect and not targeted epigenetic activity of AID likely accounts for the observed worse prognosis of patients with AID positive CLL.

## Supporting information

S1 Fig Mutational activity of AID in bacterial mutator assay. Mutational frequency is shown as ratio of Rif-resistant clones to total clones in the starting culture of UNG-deficient *E*.*coli* strain BW310 transformed with pTrc99-AID (N = 9), pTrc99-AID-GFP (N = 10) or pTrc99-GFP (N = 10). Results of individual clones are shown with mean ± SD.
(TIF)

S2 Fig AID-GFP fusion protein and GFP expression of transfected CLL samples. Cell lysates of pAID-GFP and pGFP transfected human PBMCs were assessed for protein expression of AID-GFP (51 kDa, black arrow) and GFP (27 kDa, light blue arrow) in 4 patients. Pan-Actin served as a control.
(TIF)

S3 Fig Expression of candidate genes from microarray dataset GSE39671. AICDA and five of our 45 target genes were differentially expressed in AICDA low versus high expressing samples (FDR<0.05; GABRB3, GFPT1, UNKL, GPT, DAPK1).
(TIF)

S1 Table Patient characteristics. (XLSX)

S2 Table Global methylation values in GFP and AID-GFP transfected samples using Illumina Infinium Methylation EPIC BeadChips. The methylation rate (0 = all demethylated; 1 = all methylated) for CpG sites is depicted with minimum, first quartile, median, mean, third quartile, maximum and sum values grouped according to CpG regions (ALL = all CpGs; TSS1500 = within 1500 bp upstream of the TSS; TSS200 = within 200 bp upstream of the TSS; 1stExon = within the first exon; 5UTR = within the 5′-UTR; Body = within the gene body; 3UTR = within the 3′-UTR; N Shelf = within 2–4 kb upstream of CpG islands; N Shore = within 0–2 kb upstream of CpG islands; CpG islands = within CpG islands; S Shore = within 0–2 kb downstream of CpG islands; S Shelf = within 2–4 kb downstream of CpG islands; Open Sea = isolated CpGs in the genome).
(XLSX)

S3 Table Methylation variable positions (MVPs) between paired AID-GFP and GFP samples detected by using Illumina Infinium Methylation EPIC BeadChips. (XLSX)

S4 Table Differentially methylated regions (DMRs), defined as regions with at least 3 MVPs within a region of 1kb with p<0.05, resulting in a list of 125 DMRs containing 396 MVPs. (XLSX)

S5 Table Expression analysis of microarray data GSE39671. (XLSX)

S6 Table Expression analysis of microarray data GSE22762. (XLSX)

S7 Table Correlation of AICDA expression and candidate gene expression from data GSE22762. (XLSX)
