# Supplementary figures and images for: Investigating epigenetic effects of activation-induced deaminase in chronic lymphocytic leukemia
Source: PLoS One. 2018 Dec 20;13(12):e0208753. doi: 10.1371/journal.pone.0208753 (PMC6301619; doi:10.1371/journal.pone.0208753)

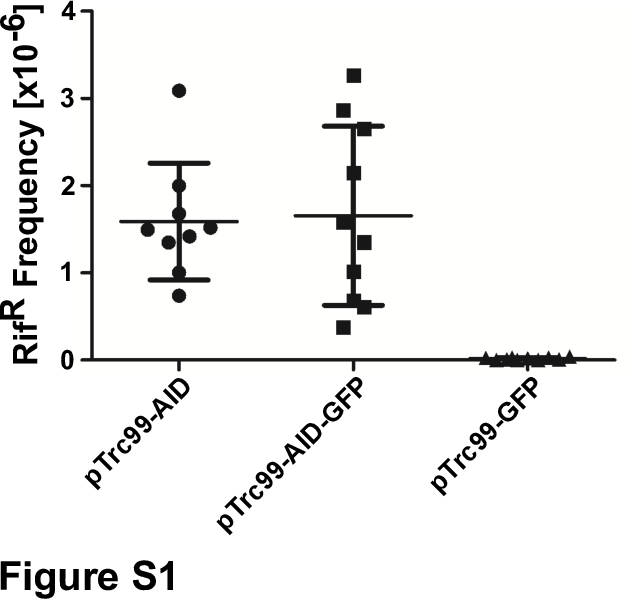

Supplement: S1 Fig — Mutational frequency is shown as ratio of Rif-resistant clones to total clones in the starting culture of UNG-deficient E.coli strain BW310 transformed with pTrc99-AID (N = 9), pTrc99-AID-GFP (N = 10) or pTrc99-GFP (N = 10). Results of individual clones are shown with mean ± SD. (TIF) [file pone.0208753.s001.tif]

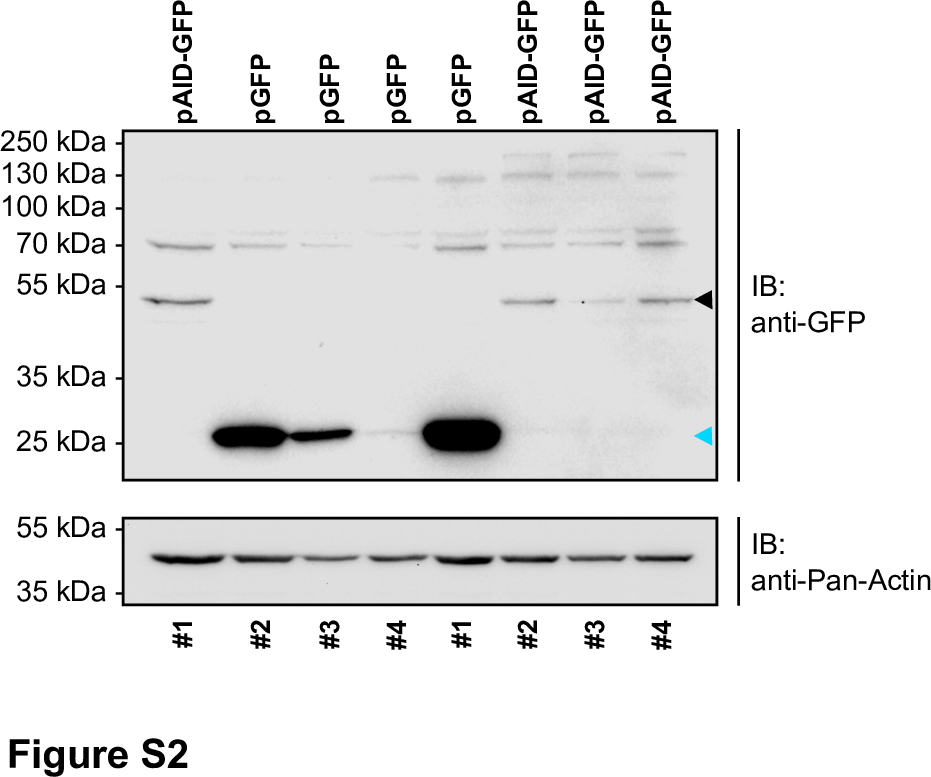

Supplement: S2 Fig — Cell lysates of pAID-GFP and pGFP transfected human PBMCs were assessed for protein expression of AID-GFP (51 kDa, black arrow) and GFP (27 kDa, light blue arrow) in 4 patients. Pan-Actin served as a control. (TIF) [file pone.0208753.s002.tif]

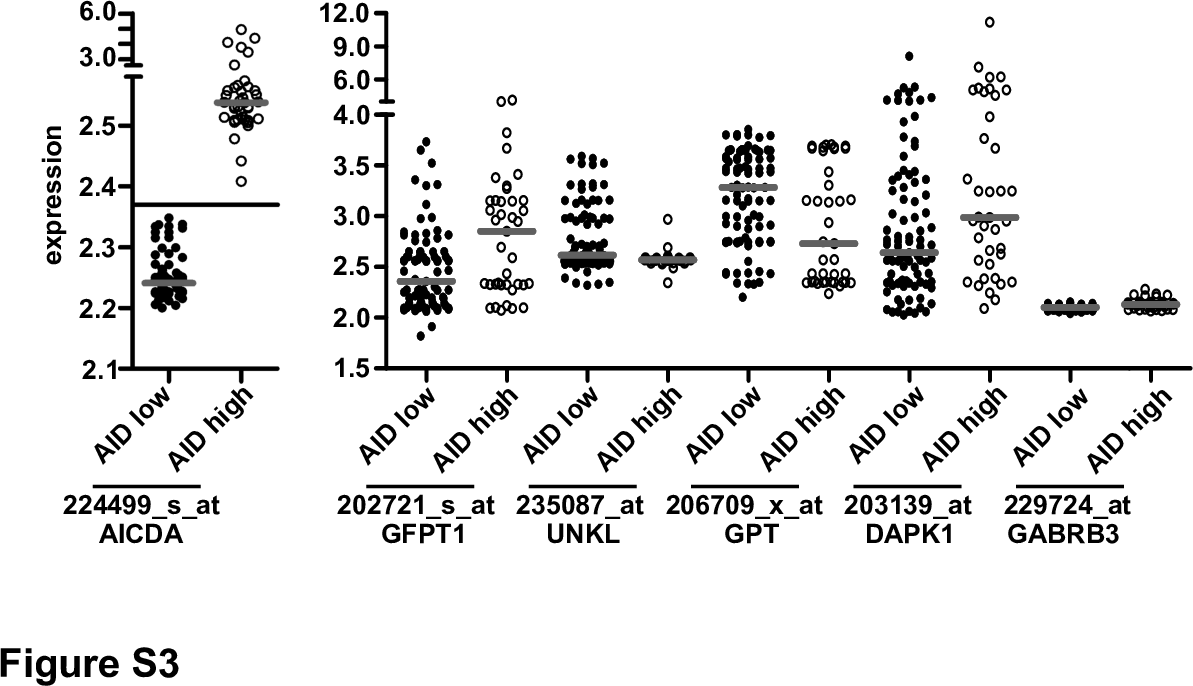

Supplement: S3 Fig — AICDA and five of our 45 target genes were differentially expressed in AICDA low versus high expressing samples (FDR<0.05; GABRB3, GFPT1, UNKL, GPT, DAPK1). (TIF) [file pone.0208753.s003.tif]
